# A nomogram model in elderly patients with coronary heart disease for predicting prognosis: research based on a real-world registry in China

**DOI:** 10.3389/fcvm.2025.1560878

**Published:** 2025-12-09

**Authors:** Wenxing Peng, Yunnan Zhang, Xiujin Shi, Yang Lin

**Affiliations:** 1 Department of Pharmacy, Beijing Anzhen Hospital, Capital Medical University, Beijing, China; 2 Department of Pharmacy, Affiliated Cancer Hospital of Zhengzhou University and Henan Cancer Hospital, Zhengzhou, Henan, China

**Keywords:** elderly patients, coronary heart disease, major adverse cardiovascular or cerebrovascular events, nomogram model, risk prediction

## Abstract

**Purpose:**

Coronary heart disease (CHD) is closely associated with aging and has become the leading cause of death in the elderly (≥65 years). This study aimed to identify independent risk factors for 2-year major adverse cardiovascular and cerebrovascular events (MACCE) in elderly patients with CHD, construct a nomogram model for predicting MACCE risk, and validate its performance to assist in identifying high-risk patients and optimizing secondary prevention strategies.

**Methods:**

Patients aged ≥65 years diagnosed with CHD were included. The primary outcome of the study was MACCE. The secondary outcomes included cardiovascular death and cardiovascular readmission. A nomogram model was constructed. Patients were divided into low-risk, medium-risk, and high-risk groups according to the tertiles of the nomogram model scores, and the primary and secondary outcomes of patients with different risks were compared.

**Results:**

This study finally included 8,340 elderly patients with CHD. MACCE occurred in 523 patients during the follow-up period, with an incidence rate of 6.3%. The Least Absolute Shrinkage and Selection Operator (LASSO) regression method was used to screen 11 independent factors associated with MACCE within 2 years. The model had a good predictive value for MACCE, with a C-statistic of 0.765 (95% CI: 0.743–0.788). The MACCE rates ranged from low risk 1.6%, medium risk 4.2% to high risk 12.6%, indicating that the nomogram model can effectively distinguish high risk patients (Log-rank *P* < 0.001).

**Conclusion:**

The established MACCE risk nomogram prediction model for elderly patients with CHD could effectively identify high-risk elderly patients with CHD.

## Introduction

With the improvement of the world economy and health levels, the proportion of the elderly population has been increasing annually. The World Health Organization predicts that by 2,050, the global population aged > 60 years will exceed 2 billion, with over 700 million people aged >75 years (over 120 million in China) (1). The growing elderly population and the remarkable rise in age-related cardiovascular diseases have resulted in a substantial surge in the prevalence of coronary heart disease (CHD) among elderly patients. Although epidemiological studies have found that dyslipidemia, diabetes, and a sedentary lifestyle are high-risk factors for cardiovascular diseases such as CHD, advanced age is undoubtedly an important risk factor (2). CHD is closely associated with aging and has become the leading cause of death among the elderly (≥65 years) (3). This study aimed to explore the risk factors associated with adverse outcomes in elderly patients with CHD, identify high-risk patients, and maximize the benefits of secondary prevention and drug therapy management.

Risk factor identification and risk stratification are prerequisites for the effective treatment and management of CHD. Clinicians and researchers are increasingly recognizing that patients classified as “extreme risk” may require special attention and intensified treatment, benefiting the most from enhanced risk factor reduction (4). Currently, risk stratification tools have been validated in clinical practice to identify high-risk patients and manage them accordingly. For example, the SYNTAX score is a scoring tool based on coronary angiography parameters that is used to guide the selection of revascularization methods for patients with coronary artery disease, such as coronary artery bypass grafting (CABG) or percutaneous coronary intervention (PCI). The Global Registry of Acute Coronary Events (GRACE) score (5) is widely used to assess the risk of death during hospitalization and within 6 months after discharge in patients with acute coronary syndrome (ACS) and is recommended for risk stratification management in patients with non-ST-segment elevation myocardial infarction (NSTEMI) (6). Thrombolysis in Myocardial Infarction (TIMI) risk score (7) and Global Use of Strategies to Open Occluded Coronary Arteries (GUSTO) risk score (8) are also commonly used to evaluate the risk of death in patients with STEMI.

Due to the presence of more risk factors and worse prognosis in elderly patients, and the fact that certain cardiovascular risk factors may have different effects in compared with younger patients, scoring models developed for the general population may not be applicable to elderly patients. For instance, high total cholesterol levels are considered a cardiovascular risk factor; however, in elderly patients (≥85 years), high total cholesterol levels are negatively correlated with the risk of death. For every 1 mmol/L increase in total cholesterol, the mortality rate decreases by 15% (9). Studies by DeFilippis et al. (10) showed that age is an important factor contributing to the overestimation of the AHA-ACC-ASCVD risk score. For each additional decade in age, the average overestimated absolute risk increases by 3.7%. Therefore, risk assessment for elderly patients requires more individualized scoring tools. The primary objective of this study is to develop and validate a user-friendly nomogram model for predicting 2-year MACCE in elderly CHD patients based on real-world registry data, addressing the lack of individualized risk assessment tools for this population.

## Methods

### Data source

This study was a retrospective analysis of data from the PHARM-Aging registry (NCT05246722). In the PHARM-Aging registry, patients were consecutively recruited from January 2019 to December 2021 if they were diagnosed with CHD at Beijing Anzhen Hospital. Medical data, including baseline and follow-up data, were recorded in an electronic data capture system (EDCs) and regularly monitored for data quality by specialized staff. The study protocol was approved by the ethics committee of Beijing Anzhen Hospital, and patient privacy was protected with the approval of corresponding regulatory agencies throughout the study.

### Study population and study design

The inclusion criteria for the study were as follows: (1) patients who provided informed consent; (2) age ≥ 65 years old; (3) patients diagnosed with CHD, CHD was diagnosed based on clinical manifestations (such as typical chest pain) combined with imaging evidence, including coronary artery CT angiography showing ≥50% stenosis in at least one major coronary artery or coronary angiography confirming coronary artery stenosis; and (4) admission for CHD, including stable angina, unstable angina, and acute myocardial infarction.

The exclusion criteria were as follows: (1) severe lack of important information such as medication history, medical history, and surgical history; (2) diagnosis of severe liver dysfunction (Child-Pugh class C); (3) severe renal dysfunction (creatinine clearance rate < 30 mL/min); (4) autoimmune diseases (systemic autoimmune diseases, organ-specific autoimmune diseases); (5) malignant tumors, multi-organ failure; (6) mental abnormalities, inability to communicate with the researchers, or inability to comply with the study protocol; and (7) in-hospital death.

### Sample size estimation

The sample size was estimated based on clinical outcomes, supplemented by estimation using the number of variables. This study intends to collect approximately 60 variables. The sample size was estimated to be 30 times the total number of risk factors, requiring at least 1,800 cases for model development. The PHARM-Aging database contains approximately 8,000 patients, which is sufficient for model establishment. Furthermore, the 2-year anticipated incidence rate of the endpoint event is approximately 7∼8%, with an estimated 600 patients experiencing the event. Consequently, the final number of independent variables in the model will not exceed 60.

### Study endpoints

The primary endpoint was defined as major adverse cardiovascular and cerebrovascular events (MACCE) within 2 years, which included all-cause death, acute ischemic stroke, and non-fatal acute myocardial infarction (MI) (11). The secondary endpoints included cardiovascular disease mortality within 2 years and cardiovascular disease readmission within 2 years.

### Follow-up

The following information was collected: clinical outcomes, changes in antiplatelet drugs, medication compliance, and adverse drug reactions. This information was collected from electronic medical records, telephone interviews, WeChat, or clinic visits, and uploaded to the EDCs.

### Statistical analysis

Statistical analyses were performed using SPSS (version 26.0) and R software (version 4.3.2). Continuous variables that followed a normal distribution were presented as mean ± standard deviation, and group comparisons were conducted using *t*-test or one-way analysis of variance (ANOVA). If the continuous variables did not follow a normal distribution, they were presented as median (with interquartile range), and group comparisons were performed using non-parametric tests. Categorical variables are presented as frequencies (percentages), and group comparisons were conducted using the chi-square test. Variable selection was performed using the least absolute shrinkage and selection operator (LASSO) regression method, implemented through the “glmnet” package in R. The occurrence of MACCE served as the dependent variable, where patients who experienced MACCE were coded as 1 and those who did not were coded as 0. Ten-fold cross-validation was used to select the optimal penalty parameter Lambda (*λ*), and Lambda + 1se was chosen to avoid model overfitting. To evaluate the model's performance, decision curve analysis (DCA) curve and receiver operating characteristic (ROC) curve were employed. Model calibration was performed using calibration curves based on 1,000 bootstrap samples. Survival analysis was performed using the Cox proportional hazards regression model. Statistical significance was set at *P*-value <0.05.

## Results

A total of 8,340 elderly patients with CHD were included in this study. The average age of the patients was 70.8 ± 4.9 years (range: 65–100 years) (Table 1). Among them, 5,137 (61.6%) were male, and 3,203 (38.4%) were female. The highest proportion of patients with CHD was diagnosed with unstable angina (UA), with 6,355 cases, accounting for 76.2% of the total. A total of 375 cases (4.5%) were diagnosed with NSTEMI, and 278 cases (3.3%) diagnosed with STEMI. Stable CHD was present in 1,332 cases (16.0%). There were 5,760 cases (69.1%) of hypertension, and 5,925 cases (71.0%) of hyperlipidemia. PCI was performed in 2,668 cases (32.0%), with a significantly lower proportion in patients with MACCE (17.8%) than in those without (32.9%, *P* < 0.001).

**Table 1:** Baseline clinical characteristics and demographic information of the enrolled patients with CHD.

Variables	All patients	Without MACCE	With MACCE	*P*
	*N* = 8,340	*N* = 7,817	*N* = 523	
Demographics				
Age (year)	70.8 ± 4.9	70.5 ± 4.7	74.5 ± 6.9	<0.001
Male, *n* (%)	5137 (61.6)	4805 (61.5)	332 (63.5)	0.360
BMI (kg/m^2^)^a^	25.3 (23.2, 27.4)	25.3 (23.2, 27.4)	24.7 (22.5, 27.1)	0.006
Systolic blood pressure (mm Hg)^a^	130 (121, 142)	130 (121, 142)	130 (119, 140)	0.001
Diastolic blood pressure (mm Hg)^a^	75 (68, 80)	75 (68, 80)	72 (65, 80)	<0.001
Heart rate (bpm)	72.6 ± 11.6	72.5 ± 11.5	74.5 ± 12.3	<0.001
Smoking, *n* (%)	1,529 (18.3)	1,446 (18.5)	83 (15.9)	0.125
Complication, *n* (%)				
Hypertension	5,760 (69.1)	5,393 (69.0)	361 (69.0)	0.571
Hyperlipidemia	5,925 (71.0)	5,593 (71.5)	332 (63.5)	<0.001
Diabetes	3,011 (36.1)	2,786 (35.6)	225 (43.0)	0.001
Heart Failure	196 (2.3)	145 (1.8)	51 (9.7)	<0.001
Prior CI	1,019 (12.2)	910 (11.6)	109 (20.8)	<0.001
Prior PCI	2,961 (35.5)	2,850 (36.5)	111 (21.2)	<0.001
Prior CABG	1,171 (14.0)	1,093 (14.0)	78 (14.9)	0.508
CHD type, *n* (%)				
SA	1,332 (16.0)	1,207 (15.4)	125 (23.9)	<0.001
UA	6,355 (76.2)	6,039 (77.3)	316 (60.4)	
Non-STEMI	375 (4.5)	324 (4.1)	51 (9.8)	
STEMI	278 (3.3)	247 (3.2)	31 (5.9)	
Intervention				
PCI	2,668 (32.0)	2,575 (32.9)	93 (17.8)	<0.001
Balloon dilation	350 (4.2)	330 (4.2)	20 (3.8)	0.661
CABG	1,161 (13.9)	1,084 (13.9)	77 (14.7)	0.584
Conservative treatment	4,161 (49.9)	3,828 (49.0)	333 (63.7)	<0.001
Examination				
LVEF (%)	60.4 (60.0, 65.0)	60.4 (60.0, 65.0)	60.0 (51.0, 64.0)	<0.001
LVDD (mm)	32.0 ± 6.3	31.7 ± 6.0	35.4 ± 9.0	<0.001
Uric acid (μmol/L)^a^	329.9 (275.6, 392.9)	328.2 (274.5, 390.3)	366.5 (295.8, 445.7)	<0.001
ALT (U/L)^a^	20 (14, 26)	20 (15, 26)	19 (13, 26)	0.006
AST (U/L)^a^	22 (18, 27)	22 (18, 27)	22 (18, 28)	0.121
BNP (pg/mL)^a^	65 (31, 165)	61 (30, 150)	199 (70, 490)	<0.001
LDL-C (mmol/L)	2.30 ± 0.81	2.29 ± 0.81	2.36 ± 0.84	0.092
HDL-C (mmol/L)	1.12 ± 0.28	1.12 ± 0.28	1.05 ± 0.29	<0.001
TC (mmol/L)	3.99 ± 0.99	3.99 ± 0.99	3.99 ± 1.02	0.998
TG (mmol/L)	1.52 ± 1.01	1.52 ± 1.02	1.43 ± 0.79	0.077
HCY (μmol/L)^a^	13.9 (11.3, 15.8)	13.1 (10.8,16.5)	15.1 (12.2,19.9)	<0.001
hsCRP (mg/L)^a^	1.23 (0.57, 3.14)	1.2 (0.56, 3.04)	2.0 (0.9, 5.1)	<0.001
FBG (g/L)	3.3 ± 0.7	3.27 ± 0.73	3.49 ± 0.90	<0.001
D-Dimer (ng/mL)^a^	145 (92, 253)	141 (90, 254)	229 (140, 353)	<0.001
Urea (mmol/L)	6.40 ± 1.90	6.27 ± 2.69	8.32 ± 4.90	<0.001
eGFR < 60 mL/min/1.73 m^2^	2,023 (24.3)	1,816 (23.2)	207 (39.6)	<0.001
Concomitant medication, *n* (%)				
Aspirin	6,742 (80.8)	6,390 (81.7)	352 (67.3)	<0.001
P2Y12 inhibitor	4,634 (55.6)	4,405 (56.4)	229 (43.8)	<0.001
ACE inhibitor/ARB	2,929 (35.1)	2,737 (35.0)	192 (36.7)	0.435
Beta blocker	5,140 (61.6)	4,804 (61.5)	336 (64.2)	0.208
Statin therapy	6,772 (81.2)	6,413 (82.0)	359 (68.6)	<0.001
PPI	5,630 (67.5)	5,284 (67.6)	346 (66.2)	0.488

CHD, coronary heart disease; BMI, body mass index; ACS, acute coronary syndrome; SA, stable angina; UA, unstable angina; STEMI, ST-segment elevation myocardial infarction; CI, cerebral infarction; PCI, percutaneous coronary intervention; CABG, coronary artery bypass grafting; LVEF, left ventricular ejection fraction; LVDD, left ventricular end diastolic dimension; ALT, alanine aminotransferase; AST, aspartate aminotransferase; BNP, B-type natriuretic peptide; LDL-C, low-density lipoprotein cholesterol; HDL-C, high-density lipoprotein cholesterol; TC, total cholesterol; TG, triglyceride; HCY, homocysteine; hsCRP, hypersensitive C-reactive protein; FBG, fibrinogen quantification; eGFR, estimate glomerular filtration rate; ACE, angiotensin-converting enzyme; ARB, angiotensin receptor blocker; PPI, proton pump inhibitor.

a Variables do not conform to normal distribution, expressed as median (quartile), and non-parametric test is used for comparison between groups.

The average follow-up time for all enrolled patients was 903.5 ± 147.9 days. During the follow-up, 523 patients experienced major adverse cardiovascular and cerebrovascular events (MACCE), with an incidence rate of 6.3%. Among them, 429 patients (5.1%) died, 13 patients (0.2%) had MI, and 84 patients (1.0%) had stroke.

**Figure 1:**
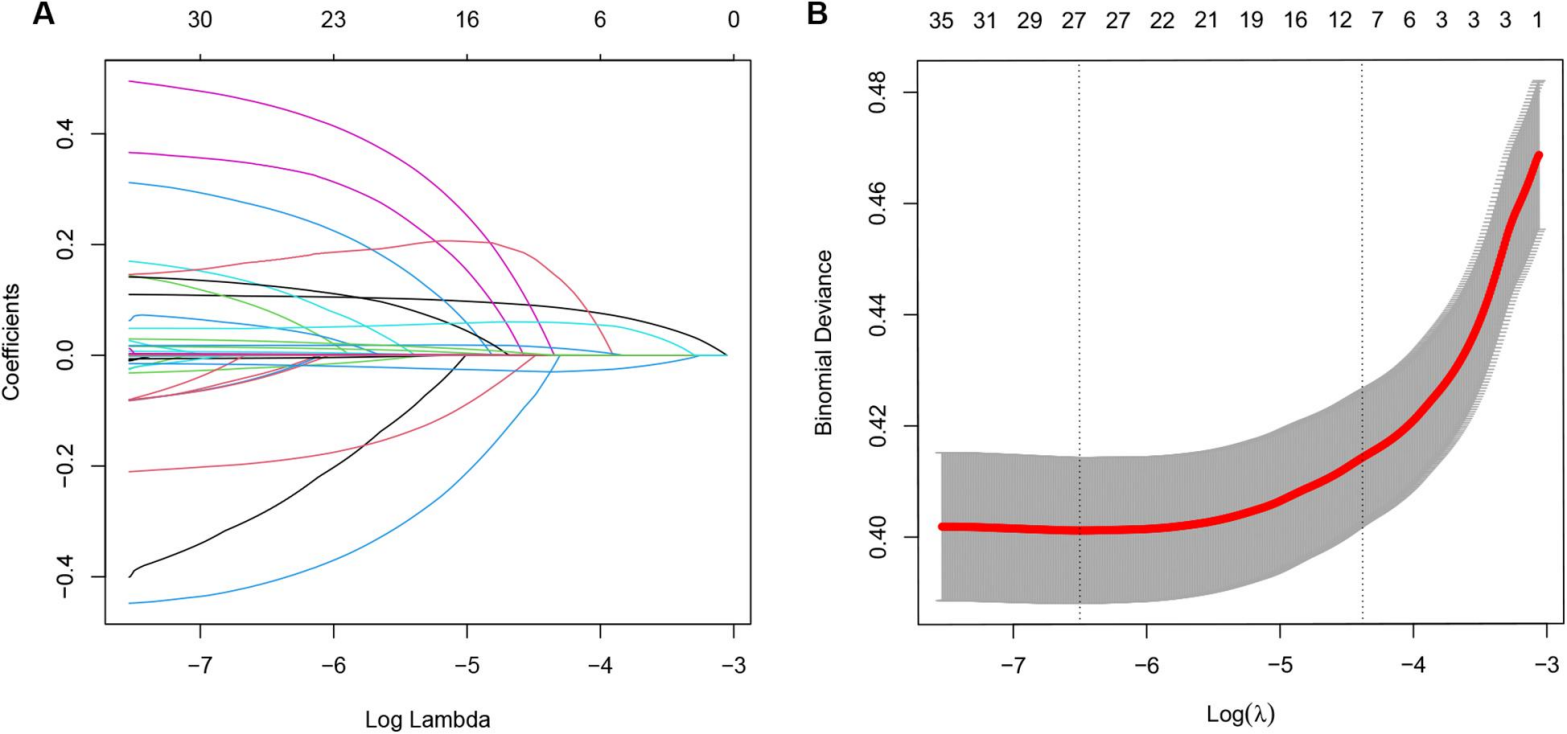
The least absolute shrinkage and selection operator (LASSO) regression was used to extract features. **(A)** The results showed that 27 variables were retained at the point where the error was the smallest, which is represented by the position of the dotted line on the left **(B)** To avoid overfitting and simplicity of the model, only variables within one standard error (1se) of the minimum error were selected, and 11 variables were retained, which corresponded to the place on the dotted line on the right **(B)**.

**Figure 2:**
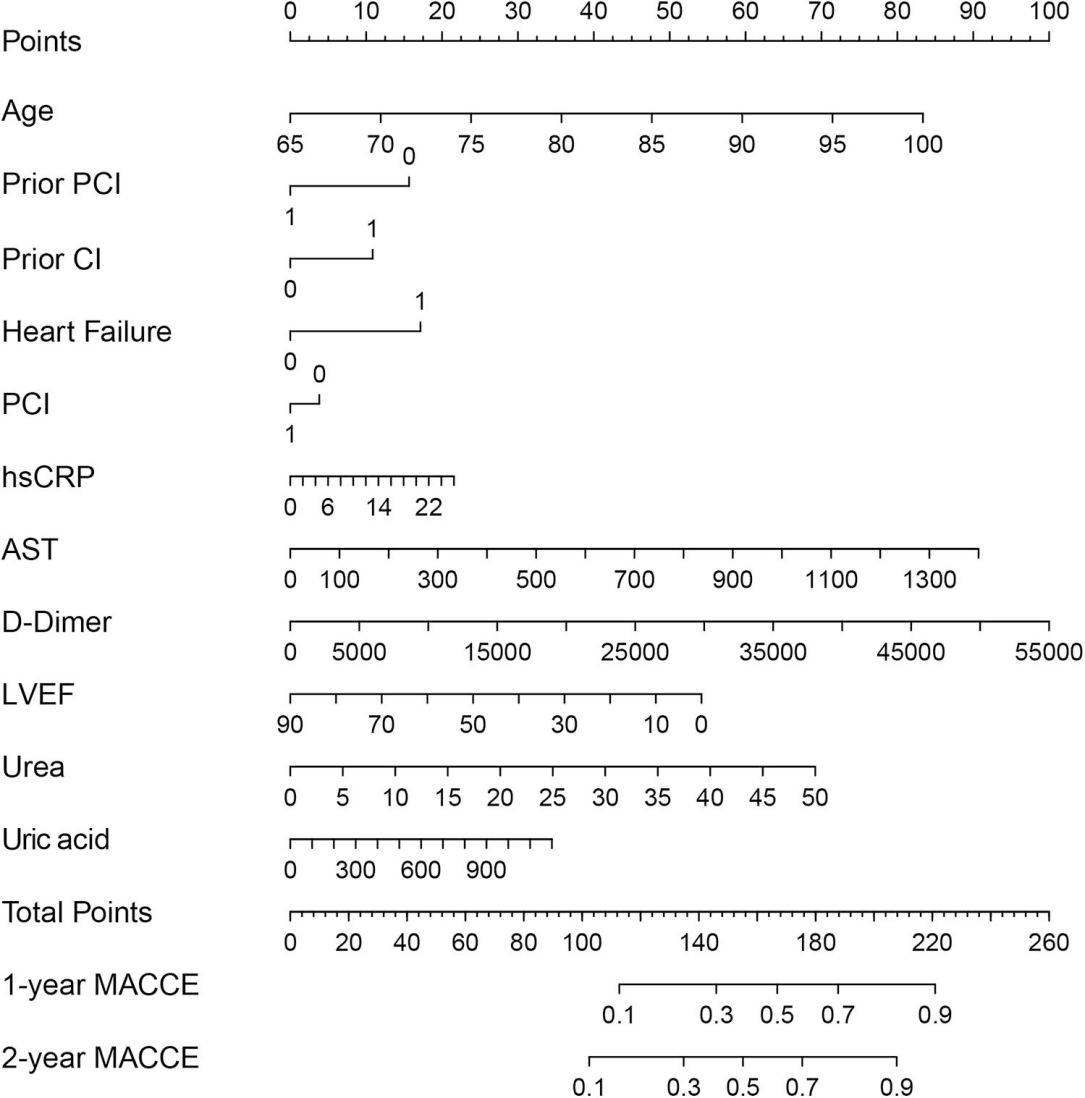
MACCE risk nomogram model in elderly patients with CHD. The model showing a scoring system for predicting cardiovascular risks. It includes variables like age, prior PCI, prior CI, heart failure, PCI, hsCRP, AST, D-Dimer, LVEF, urea, and uric acid. Points are calculated and total points relate to the probability of 1-year and 2-year MACCE. MACCE, major adverse cardiac and cerebrovascular events; CHD, coronary heart disease; PCI, percutaneous coronary intervention; CI, cerebral infarction; hsCRP, hypersensitive C-reactive protein; AST, aspartate aminotransferase; LVEF, left ventricular ejection fraction.

Variable selection was performed using LASSO regression method. Figure 1A displays the variables selected by LASSO regression, while Figure 1B indicates the optimal lambda + 1se position and the 11 variables identified. The results revealed that the risk factors associated with MACCE included age, prior PCI, prior cerebral infarction (CI), heart failure, PCI, uric acid, aspartate aminotransferase (AST), hypersensitive C-reactive protein (hsCRP), D-Dimer, left ventricular ejection fraction (LVEF), and urea. These factors are detailed in Table 2. The 11 significant variables from the LASSO regression model, including age, prior PCI, prior CI, heart failure, PCI, uric acid, AST, hsCRP, D-Dimer, LVEF, and urea were incorporated into the nomogram model. Figure 2 shows the nomogram model, which represents the contribution of each variable to the outcome of the scoring system. The length of each segment corresponded to the score assigned to each factor. The sum of the scores for the 11 variables represents the patient's risk score, with the corresponding 1 or 2-year MACCE risk shown at the bottom of the nomogram model.

**Table 2:** Multivariable analysis of risk factors associated with MACCE by LASSO regression.

Variables	Risk factors	Coefficient
X1	Age (year)	0.006122
X2	Prior PCI	0.001770
X3	Prior CI	0.039466
X4	Heart Failure	−0.002121
X5	PCI	0.000049
X6	Uric acid (μmol/L)	0.001025
X7	hsCRP (mg/L)	0.000029
X8	AST (U/L)	0.000002
X9	D-Dimer (mg/L)	−0.002221
X10	LVEF (%)	0.005630
X11	Urea (mmol/L)	0.006122
Intercept	–	−0.295268

LASSO, the least absolute shrinkage and selection operator; PCI, percutaneous coronary intervention; CI, cerebral infarction; hsCRP, hypersensitive C-reactive protein; AST, aspartate aminotransferase; LVEF, left ventricular ejection fraction;.

**Figure 3:**
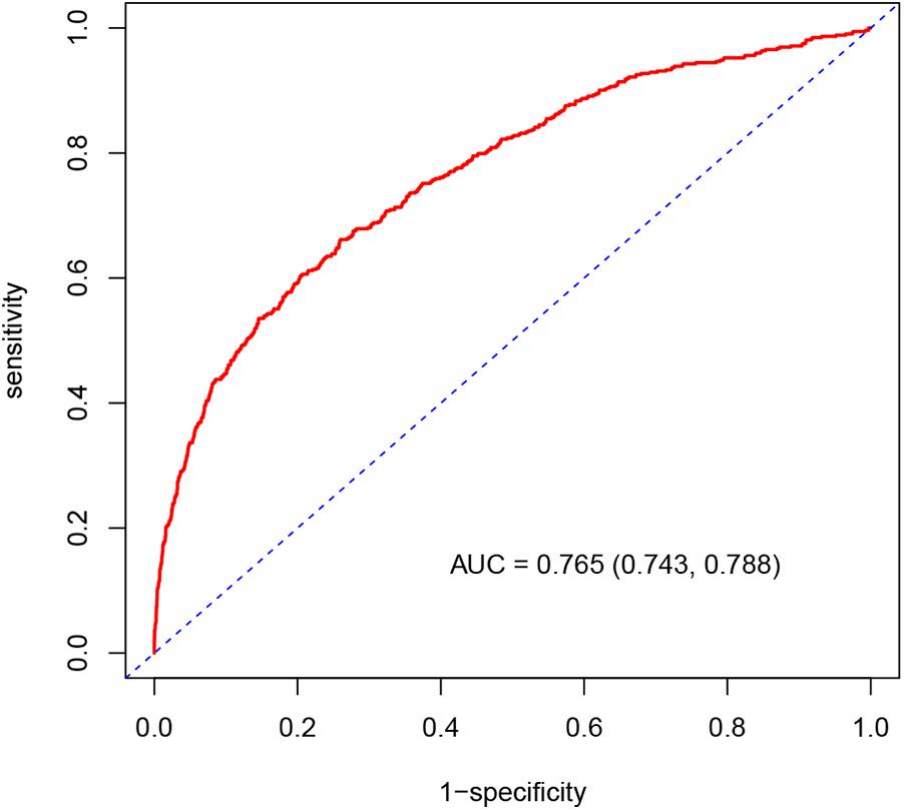
ROC curve of MACCE risk nomogram model in elderly patients with CHD. ROC curve, receiver operating characteristic curve; AUC, area under the curve; MACCE, major adverse cardiovascular or cerebrovascular events; CHD, coronary heart disease.

**Figure 4:**
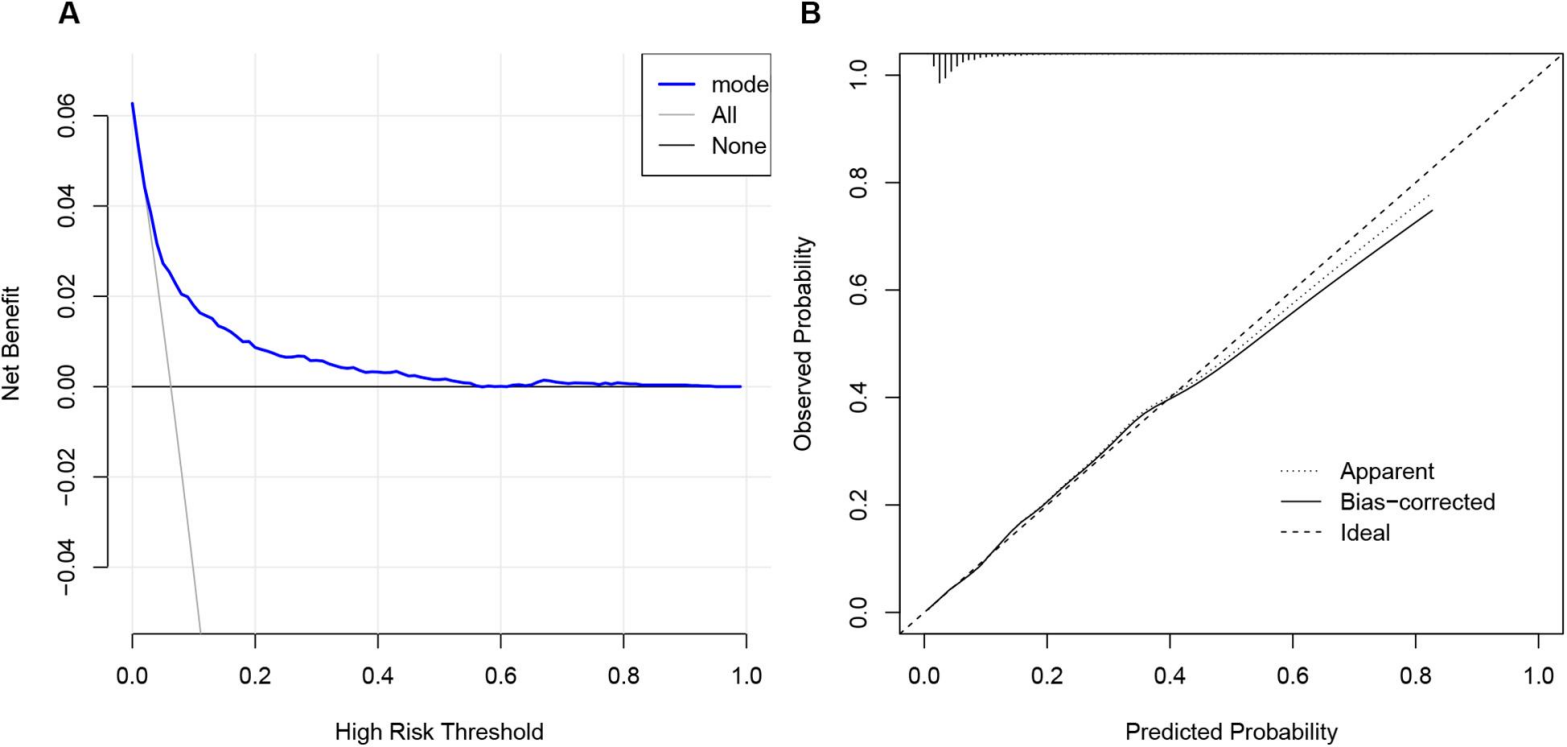
Decision curve analysis **(A)** and calibration curve of nomogram model for MACCE risk in elderly patients with CHD **(B)**. MACCE, major adverse cardiovascular or cerebrovascular events; CHD, coronary heart disease.

The ROC curve of the established nomogram model for MACCE risk in elderly CHD patients is shown in Figure 3. The C-statistic of the model was 0.765 [95% confidence interval (CI): 0.743–0.788]. DCA analysis was performed to assess the clinical utility of the model, as depicted in Figure 4A, and calibration curves were plotted to evaluate the consistency between the predicted and observed outcomes, which indicated good calibration of the model (Figure 4B).

**Figure 5:**
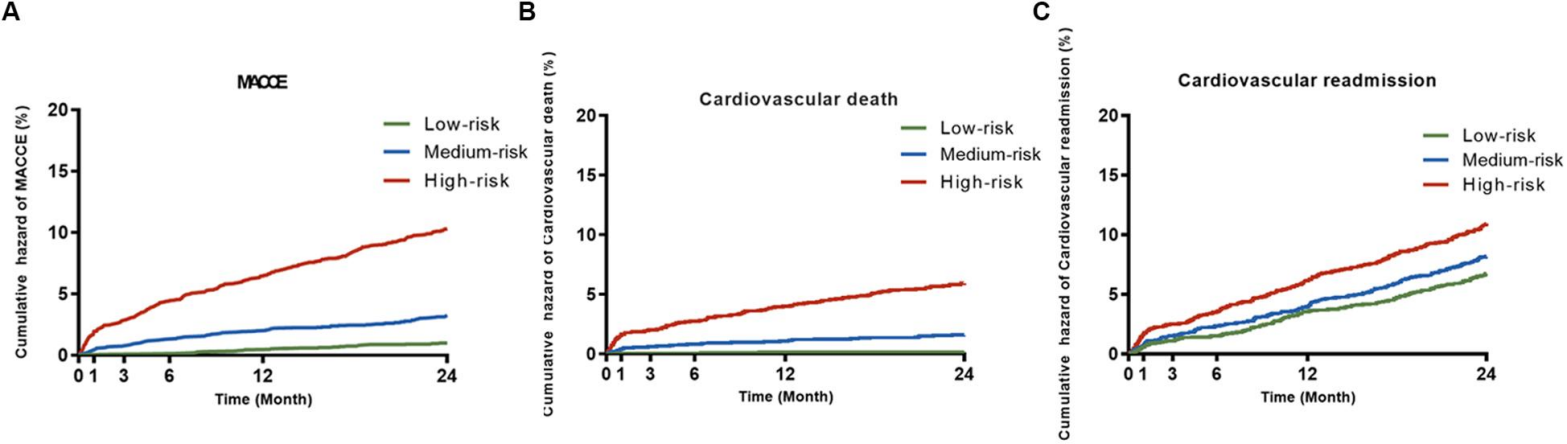
Kaplan–meier (K–M) survival curves of patients with different risks. **(A)** MACCE; **(B)** cardiovascular death; **(C)** cerebrovascular readmission. MACCE, major adverse cardiovascular or cerebrovascular events.

Based on the third quartile score of the nomogram model, patients were classified as low-risk (0–59 points) with 2,698 cases, medium-risk (60–77 points) with 2,815 cases, and high-risk (≥78 points) with 2,827 cases. The 2-year MACCE rates in the low, medium, and high-risk groups were 1.6%, 4.2%, and 12.6%, respectively. There was a significant difference in event rates among the groups (Log-rank *P* < 0.001). Further investigation revealed that the nomogram model effectively differentiated the risk of cardiovascular disease mortality and cardiovascular disease readmission. The comparison of cardiovascular disease mortality and cardiovascular disease readmission rates among the low-risk, medium-risk, and high-risk groups all showed statistically significant differences (Log-rank *P* < 0.001), and the detailed results are shown in Figure 5.

## Discussion

This study was based on real-world cohort data of 8,340 elderly patients with CHD. The LASSO regression method was applied to select 11 independent factors associated with MACCE within 2 years, including age, prior PCI, prior CI, heart failure, PCI, uric acid, AST, hsCRP, D-Dimer, LVEF, and urea. These 11 variables were included in the nomogram model, showing a good predictive value with a C-statistic of 0.765 (95% CI: 0.743–0.788). Patients were divided into high, medium, and low-risk categories based on the tertiles of the nomogram score, and MACCE rates varied from 1.6% in the low-risk group to 4.2% in the medium-risk group and 12.6% in the high-risk group. The results indicated that the nomogram model could effectively identify high-risk elderly CHD patients.

Although the implementation of various strategies for the prevention and treatment of CHD has significantly reduced cardiovascular mortality in recent decades, it remains one of the diseases with the highest incidence and mortality worldwide. Risk factor identification and stratification are prerequisites for the treatment and management of cardiovascular diseases. High-risk patients may benefit more from early and aggressive treatment interventions, improving their clinical prognosis. Previous studies have developed CHD risk prediction models, such as SYNTAX score (12), EuroHeart score (13), TIMI risk score (7), GRACE risk score (5), CADILLAC risk score, and TRS2P score (14). However, due to limitations in the study population and treatment modalities of the model development cohorts, the established models may not be fully applicable to contemporary clinical practice, resulting in limitations in their use and potential errors in risk estimation (10). These limitations include: (1) most scores are based on European and American populations, which may not be fully applicable to Chinese patients due to population differences; (2) except for the GRACE score, most scores are based on data from large randomized controlled trials, and the predictive value of derived models in routine clinical practice needs to be validated; (3) the development cohort of the GRACE model had only 26.6% of ACS patients receiving PCI, and the CADILLAC risk score model's development cohort used bare-metal stents. Additionally, in the era of reperfusion therapy, statins, and antiplatelet drugs are widely used. These differences in treatment modalities may render the previously developed models less applicable to contemporary clinical practice; (4) most previous models were used for short-term treatment decisions and prognosis risk assessment, and there are fewer models that evaluate the long-term prognosis risk of CHD patients; (5) due to the completely different roles of some risk factors in elderly patients compared with younger patients, risk assessment for elderly CHD patients requires more individualized scoring tools.

Several existing nomograms have been developed for cardiovascular outcome prediction in elderly or ischemic heart disease (IHD) patients. Chen et al. (15) developed a PCI-specific nomogram for elderly patients to predict 2-year and 5-year target vessel revascularization, with diabetes, post-PCI quantitative flow ratio (QFR), prior MI, and prior PCI as predictive variables [area under the curve (AUC): 0.742–0.789]. In contrast, our model targets all elderly CHD patients (not just PCI recipients) and predicts MACCE with a C-statistic of 0.765 (95% CI: 0.743–0.788), expanding applicability. Notably, our real-world data showed that PCI was performed in 2,668 (32.0%) cases, with a significantly lower proportion in patients with MACCE (17.8%) than in those without (32.9%, *P* < 0.001), suggesting that performing PCI may be a protective factor for elderly CHD patients. Yang et al. (16) constructed an IHD mortality nomogram (1/3/5-year C-index: 0.658–0.739) using age, uric acid, and liver/cardiac function biomarkers. While sharing age/uric acid as predictors, our model includes more diverse variables and focuses on MACCE (not just mortality), enabling comprehensive risk stratification. Our model also effectively differentiates 2-year MACCE rates across risk groups and cardiovascular death/readmission risks, supporting holistic secondary prevention in elderly CHD patients.

Several prospective cohort studies have indicated that hyperuricemia is an independent risk factor for hypertension, diabetes, coronary artery disease, and stroke (17–19). Luca et al. (20) evaluated the impact of uric acid levels on major adverse cardiovascular events in patients with chronic coronary artery syndrome and found that patients with high uric acid levels had a higher risk of major adverse cardiovascular events (including cardiovascular mortality, hospitalization for MI, heart failure, angina or revascularization) than those with low uric acid levels. Han et al. (21) reported that CRP is risk factor for coronary artery stenosis in elderly patients with CHD, and CRP level is positively correlated with the severity of coronary artery lesions. D-Dimer, as a degradation product of fibrin, indicates the presence of a hypercoagulable state and secondary fibrinolysis. Kikkert et al. (22) investigated the predictive value of D-Dimer for major adverse cardiovascular events (including all-cause death, recurrent MI, stroke, or target vessel revascularization for ischemia) in patients with acute myocardial infarction (AMI) and found that D-Dimer levels ≥0.71 μg/mL upon admission were risk factors.

Although hyperlipidemia was not identified as an independent risk factor for MACCE in the multivariate regression analysis, we found a significantly higher proportion of hyperlipidemia in patients without MACCE than those with MACCE when comparing the baseline data. This finding may be inconsistent with those observed in younger patients. A previous study showed that, although high total cholesterol levels are considered cardiovascular risk factors, in elderly patients (≥85 years old), higher total cholesterol levels were negatively correlated with the risk of death. Each 1 mmol/L increase in total cholesterol was associated with a 15% decrease in mortality. One possible explanation is that higher cholesterol levels are associated with lower cancer levels and are negatively correlated with nosocomial infections, which are the main causes of death in elderly patients (9).

The main limitations of this study are as follows: (1) It was a single-center study with internal validation only, which may affect the generalizability of the model. Future improvements and validation of the model would require multi-center cohorts. (2) Due to the limitations of observational research, additional variables that may be related to outcomes, such as genotypes and other serum biomarkers, were not obtained. (3) Considering the convenience and usability of the model, the evaluation of CHD severity using coronary artery calcification scores was not included.

## Conclusions

The established nomogram model for elderly patients with CHD can effectively predict the risk of MACCE and identify high-risk elderly patients with CHD.

## Data Availability

The raw data supporting the conclusions of this article will be made available by the authors, without undue reservation.
